# Challenges of blinding in clinical balneology trials: a scoping review

**DOI:** 10.1186/s12906-025-04878-y

**Published:** 2025-04-11

**Authors:** Katalin Szendi, Szimonetta Lohner, Ágnes Szenczi, Edit Murányi, Károly Berényi, Balázs Németh

**Affiliations:** 1 Department of Public Health Medicine, Medical School, University of Pécs, Pécs, Hungary; 2 Department of Public Health Medicine, MTA–PTE Lendület “Momentum” Evidence in Medicine Research Group, Medical School, University of Pécs, Pécs, Hungary

**Keywords:** Balneology, Scoping review, Blinding, Medicinal water, Mineral water, Clinical trial

## Abstract

**Background:**

In evidence-based medicine, randomized, placebo-controlled, double-blind clinical trials are considered the ‘gold standard’ of study design. Efforts must be made to advance evidence-based balneology in a similar manner. The objective of this scoping review was to assess the intervention types of experimental and control groups used in clinical balneology trials to map the proportion of open-label, single- and double-blind studies.

**Methods:**

Eligibility criteria: i) prospective interventional clinical trial, ii) focused on the therapeutic effect of natural medicinal and mineral water, iii) administered head-out immersion, iv) compared with any other intervention or no treatment, v) in adult patients, and vi) with no restrictions on study design or language. Two authors independently searched the Medline, Embase and Cochrane databases for trials published in any language between 1990 and 12 February 2025.

**Results:**

The 109, included trials were categorized into eight groups according to the treatment of the experimental and control groups and the use of blinding. Studies in the lower categories (1, 2, 3) completely lack blinding. In categories 4a-b-c, tap water control was used in parallel with medicinal/mineral water. Category 4c was the first category where the ‘gold standard’ of evidence-based medicine was implemented. Finally, in the last two categories (5a-b), validated placebo water was used. Low-category papers constituted the largest group, accounting for 74% of the total number of publications. From 1990 to the present, only 11% of publications chose the double-blind setup. Most higher category papers were published in Hungary. Over time, there has been no clear improvement in study design.

**Conclusions:**

Future balneological research should prioritize rigorous experimental designs, particularly by incorporating validated placebo water and double-blind methodologies. Without these improvements, the ability to draw reliable conclusions about the true efficacy of balneotherapy remains limited.

**Registration:**

The scoping review protocol was registered prospectively in OSF registries (Registration DOI https://doi.org/10.17605/OSF. IO/XHS4B, internet Archive link https://archive.org/details/osf-registrations-xhs4b-v1, Date registered June 26, 2022).

**Supplementary Information:**

The online version contains supplementary material available at 10.1186/s12906-025-04878-y.

## Background

Balneotherapy involves the therapeutic use of natural mineral waters, Peloids, and natural gases. This scoping review focuses exclusively on natural mineral and medicinal waters.

In this scoping review ‘mineral medicinal water’ is defined as naturally or artificially sourced mineral water with proven therapeutic effects [1]. In some countries, natural mineral waters are categorized by legal regulation on the basis of scientific data. In Hungary, the designation 'medicinal water' is regulated by law [2] and authorized by the National Public Health and Medical Officer Service, Directorate General for Spas (OGYFI). In Germany, the therapeutic effectiveness of medicinal water must be clinically proven and confirmed by the German Federal Institute for Drugs and Medical Devices [3]. In Spain, mineral-medicinal waters are defined as waters that can be used for therapeutic purposes and have been officially declared to be of Public Utility [4]. In China, waters with a certain mineral content are classified as medicinal waters on the basis of the water quality standards for balneotherapy published in the Geological Exploration Standard for Geothermal Resources (GB-T-11615–2010) [5]. Collecting publications exclusively on natural ‘mineral medicinal waters’ (with proven healing effects) is a challenging task. Many publications, possibly due to uncertain or nonexistent regulations on healing effects, do not use the term ‘medicinal/healing water’ or reference its proven therapeutic benefits. Additionally, the scientific literature on balneotherapy lacks unified terminology for natural medicinal waters. Instead, terms such as balneotherapy, spa therapy, spa water, mineral water, thermal/mineral water, and even hydrotherapy are used [6]. In Hungary, several medicinal waters have distinct characteristics, such as specific color, pH, scent, and consistency, due to the presence of sulfur and other organic substances. These features may also be found in thermal/mineral waters in other countries, but the authors have found limited information on this topic.

In evidence-based medicine (EBM), randomized, placebo-controlled, double-blind clinical trials are considered the ‘gold standard’ of study design [7, 8]. Efforts must be made to advance evidence-based balneology in a similar manner [9]. Blinding is a critical methodological feature of randomized controlled trials (RCTs) [10], especially when the patient’s or the investigator’s expectations can affect the outcome of balneotherapy. If patients know which treatment they are receiving, the placebo effect can significantly influence the results, potentially leading to false conclusions [11]. Researchers may attempt to mask the various physical and chemical properties of medicinal water or tap water by using coloring, pH adjustments, scent imitation, or providing the same environment. However, there is still a chance that experienced patients can distinguish between them. In these situations, achieving effective blinding is more challenging than simply using a white pill, as is commonly done in pharmaceutical research.

In clinical balneology trials, meta-analyses have revealed that high heterogeneity is a significant problem in many areas [9, 12–15]. Ma et al. (2021) argued that studies also exhibit methodological heterogeneity [14]. Bai et al. (2019) suggested the use of higher-quality RCTs with a double-blind design [9]. Additionally, they reported that randomization was often unclear and that the use of a double-blind design was insufficient in most studies. According to Bender et al. (2014), the only common feature of the analyzed studies was the primary outcome measure [12]. They also noted that masking control tap water was often impossible. If the discipline of balneology does not meet the requirements of evidence-based medicine, it could face funding difficulties, as health insurance companies may refuse to finance balneotherapy treatments.

The objective of this scoping review is to assess the intervention types of experimental and control groups used in clinical balneology trials to map the proportion of open-label, single- and double-blind studies. This review primarily focuses on the challenges of blinding in clinical balneological trials and proposes solutions to address them.

Considering the findings above, the following research questions arise that can be considered in a scoping process: How many and what types of interventional study designs have been used in clinical balneology? Did the proportion of single- and double-blind studies improve between 1990 and 2025?

## Methods

### Protocol and registration

Our protocol was drafted using the Preferred Reporting Items for Systematic Reviews and Meta-analysis Protocols for Scoping Reviews (PRISMA-ScR). The PRISMA-ScR checklist is provided as Additional file 1. A scoping review was used to compile all relevant evidence provided by intervention studies in balneology conducted between 1990 and 2025. The scoping review protocol was registered prospectively in OSF registries (Registration DOI https://doi.org/10.17605/OSF. IO/XHS4B, internet Archive link https://archive.org/details/osf-registrations-xhs4b-v1, Date registered June 26, 2022).

### Eligibility criteria

To be included in the scoping review, a study needed to meet all of the following criteria: i) a prospective interventional clinical trial, ii) conducted in the field of balneology; iii) focused on the therapeutic effect of natural mineral-medicinal waters and natural mineral waters; iv) administered as head-out immersion (where one’s entire body except for the head is submerged); v) compared with any other intervention or no treatment; vi) in adult patients (aged over 18 years) who are either healthy or have common musculoskeletal (e.g. knee/hip/hand osteoarthritis, low back pain, rheumatoid arthritis, fibromyalgia, persistent lumbar pain syndrome, chronic neck, shoulder and lumbar pain, spondylosis, ankylosing spondylitis, subacute supraspinatus tendinopathy, musculoskeletal pains, psoriatic arthritis, osteoporosis etc.), dermatological (e.g. psoriasis, atopic dermatitis), or gynecological conditions (mastalgia, chronic inflammatory gynecological disorders); vii) with results published between 1990 and 2025; and viii) with no restrictions on study design or language. The start date of this systematic search was 1990 because the birth of EBM occurred during that decade. This scoping review aimed to assess the most common conditions treated with balneotherapy in Hungary [12], and these musculoskeletal, dermatological, and gynecological disorders are more common in adults.

The authors chose to exclude spa therapy because it included balneotherapy, aquatic exercise, massage, mud/peloid therapy, sauna, diet, patient education, etc. Without proper control, the effect of this complex treatment was difficult to test. Other complex treatments, where the individual effects of medicinal/mineral water cannot be assessed, were also excluded. Studies in which the effects of water temperature and radon were investigated and where medicinal water treatment occurred after surgery were not considered in this review.

### Search strategy

Ovid MEDLINE, EMBASE and the Cochrane Central Register of Controlled Trials (CENTRAL) were searched from 1990 to 9 May 2022. Text words with appropriate truncation and relevant indexing terms for “medical water/balneotherapy” were used. The language of publication was not a limiting factor during research collection. The electronic searches were updated on 27 June 2023 and 12 February 2025. The search strategies were developed and executed by an experienced coauthor [Szimonetta Lohner] and further refined through team discussion. The final search strategy for Ovid MEDLINE can be found in Table 1. The final search results were exported into Covidence online software (Veritas Health Innovation, Covidence systematic review software. Melbourne, Australia), and duplicates were removed.

**Table 1:** Search strategy for Ovid MEDLINE

Database: Ovid MEDLINE(R) ALL < 1946 to June 26, 2023 >
**Search strategy:**
**1** Baths/ (5475)
**2** Mineral Waters/ (4373)
**3** balneo*.mp. (6639)
**4** healing water*.mp. (48)
**5** medical water*.mp. (20)
**6** medicinal water*.mp. (60)
**7** or/1–6 (15,040)
**8** exp animals/ not humans.sh. (5,134,178)
**9** 7 not 8 (14,316)
**10** limit 9 to yr = “1990 -Current” (5696)

Search date: 27.06.2023

### Screening and data extraction

Each reference was screened by two independent reviewers on the basis of predefined inclusion criteria via Covidence online software (Veritas Health Innovation, Covidence systematic review software. Melbourne, Australia). First, the titles and abstracts of the studies were screened to exclude irrelevant references. Second, the full texts of potentially relevant studies were checked for final inclusion.

One reviewer (KSz) developed the data-charting form to determine which variables to extract. Two reviewers (SzK, ME) independently charted the data, discussed the results, and continuously updated the data-charting form. Items were selected for charting in MS 365 Excel.

The following data were extracted from each included study by one reviewer and checked for accuracy by a second reviewer: bibliographic details (title, authors, journal, country, language, year), water parameters, and characteristics of the experimental and control groups (type of intervention, blinding, randomization) (Additional file 2) [16–124].

### Critical appraisal of individual sources of evidence

There are several assessment tools available to evaluate the quality of clinical trial reports. However, in the realm of medicinal water research, if the experimental design is flawed from the outset, the trials’ randomization, double-blinding, and precise data handling become irrelevant. A faulty initial concept will yield inaccurate data regardless of the rigor of other parameters, largely owing to the significant influence of the placebo effect on the results [125]. Therefore, the authors aimed not to classify articles on the basis of existing scores but rather to determine the place of balneology within EBM by analyzing the experimental designs, particularly the treatment of experimental and control groups. This approach aims to help researchers studying the effects of balneotherapy avoid repeating past shortcomings related to blinding.

### Synthesis of results

Data were collected on the experimental setup, language of publication, authors’ nationality, years of publication, popular journals, existence of blinding and number of publications on medicinal mineral water. Items were selected for charting in MS 365 Excel. The results are summarized in bar charts and are also described narratively.

## Results

### Selection of sources of evidence

**Fig. 1:**
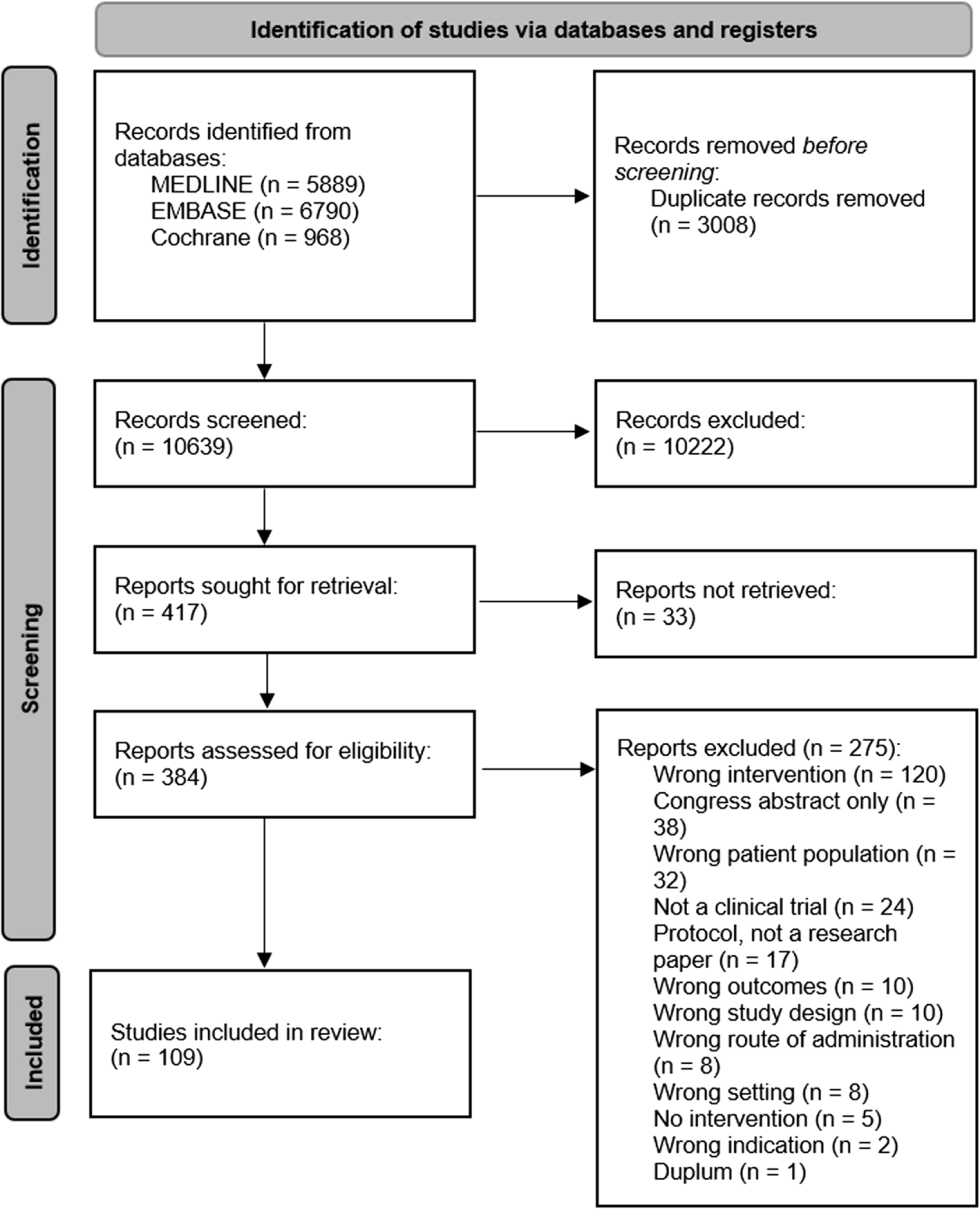
PRISMA 2020 flow diagram for new systematic reviews

Figure 1 shows the selection process using a PRISMA flow diagram.

### Characteristics of sources of evidence

Study characteristics are indicated in Additional file 2, which contains data on the name of the first author, year, country, language of publication, journal, experimental and control group treatment, water parameters, masking, randomization, category and citation.

### Results of individual sources of evidence

The included papers provided insufficient information on whether studies tested “mineral medicinal waters” with proven health effects; therefore, the articles were classified into three groups according to the scientifically (and/or legally) proven healing properties of the waters: 1) natural mineral-medicinal waters, 2) natural mineral waters, and 3) the Dead Sea.

The included papers were classified into 8 groups on the basis of the intervention of experimental and control groups and blinding (1–2-3-4a-4b-4c-5a-5b). (Information on each category is available in Table 2). Studies in the lower categories (1, 2, 3) completely lack blinding. In categories 4a, 4b (medium category) and 4c, tap water control was used in parallel with medicinal/mineral water. Category 4c (high category) was the first category where the “gold standard” of EBM (randomized, controlled, double-blind clinical trial) was implemented. Finally, in the last two (high) categories (5a and 5b), validated placebo water [125] was used.

**Table 2:** Categories of intervention setups for balneotherapy research

Experimental group	Control group and category	Blinding
BT	**1.** no control group	no
BT	**2.** other treatment/no treatment	no
BT + other treatment^a^	**3.** other treatment^a^	no/incorrect
BT (+ other treatment^b^)	**4a.** tap water thought to be placebo (+ other treatment^b^)	no/incorrect
	**4b.** tap water thought to be placebo (+ other treatment^b^)	single
	**4c.** tap water thought to be placebo (+ other treatment^b^)	double
BT (+ other treatment^b^)	**5a.** validated placebo water (+ other treatment^b^)	double
BT + no regular medication	**5b.** validated placebo water + no regular medication	double

*BT* Balneotherapy

^a^Obligatory for both groups simultaneously

^b^Optional for both groups simultaneously

Definition of “other treatment”: A new nonbalneological intervention (physiotherapy, exercise, physical activity, medicine, UVB, relaxation, hiking, education) was administered.

In both groups (the experimental and the control groups), all patients with a preexisting health condition were allowed to take their regular medications (pain killers, anti-inflammatory drugs, antirheumatic drugs, etc.). Therefore, the information on regular medication was omitted from Table 2 except in category 5b.

#### Detailed explanation of categories

According to the authors, the ideal setup of an experimental-control group in all categories would have been the one described in category 5b. On the basis of the previously mentioned category, the outcome (difference between the effects of balneotherapy [BT] and placebo water) can be measured if validated placebo water [125] is used in the control group and if the trial is at least double-blind. However, it is important to note that category 5b has a limitation. The fact that patients in experimental or control groups did not receive any additional therapy for their symptoms (e.g., pain, skin symptoms) could raise ethical concerns. Therefore, in most cases, category 5a [105] would have been the correct and best available choice to solve the existing methodological problems in lower categories. However, in category 5a, only additional effects of BT could be measured compared with those of regular and/or other treatments. Taking into account the proper setup of experimental-control groups, the categories created by the methodological problems and defined by the authors are discussed below.

Category 1 (without a control group) [16, 33–37, 44, 51, 53, 54, 60, 63, 72, 82, 86, 88, 98, 99, 109, 115, 119].

Aim of studies: To assess the improvement caused by BT compared with baseline values.

Limitations: Without blinding, the question arose whether BT improved patients’ symptoms or whether they would have improved without BT. This may be because only the patients’ faith in BT cured them. Without proper control, it is not clear whether BT is more effective than the placebo effect (= belief).

Category 2 [19, 20, 28, 31, 32, 45, 48, 56, 59, 66, 75, 90, 91, 103, 104, 110, 111, 118, 121].

Aim of studies: To assess the differences between the effects of BT and those of other treatments or no treatment.

Limitations: As blinding is not possible, there is no placebo control. Therefore, the impact of patients’ belief in BT or other treatments cannot be eliminated. In other words, two placebo effects are compared in the study.

Category 3 [18, 24, 27, 30, 38–42, 46, 47, 50, 52, 55, 64, 65, 67, 68, 71, 73, 74, 78, 79, 83–85, 90, 93–97, 100, 102, 107, 108, 112–114, 120, 122].

Aim of the studies: To assess the additional effect of BT compared with that of the other treatments that both groups received.

Limitations: As blinding is not possible, there is no placebo control. Therefore, the impact of patients’ belief in BT and other treatments together cannot be eliminated.

Category 4a (tap water in the control group) [23, 25, 26, 29, 43, 57, 58, 69, 70, 81, 87, 101, 106, 117].

Aim of studies: To assess the differences between the effects of BT and tap water.

Limitations: As the clinical trial is not blinded, patients know what they are bathing in. Therefore, the influence of belief cannot be eliminated.

Category 4b (single-blind setup in the trial with control tap water use) [92, 124].

Aim of studies: To assess the differences between the effects of BT and tap water.

Limitations: Tap water is not a validated placebo. To ensure that patients do not know which water they are bathing in, a pretest is needed [125].

Category 4c (double-blind setup in the trial with control tap water use) [17, 21, 22, 49, 61, 62, 76, 77, 80, 116, 123].

These studies have the same limitations as those in category 4b.

On the basis of the extracted data, the following results were obtained.

Most of the articles were written in English (88.07%). Among the 109 articles, 5 were written in German, 4 were written in Japanese, 3 were written in Turkish, and 1 was written in Italian.

**Fig. 2:**
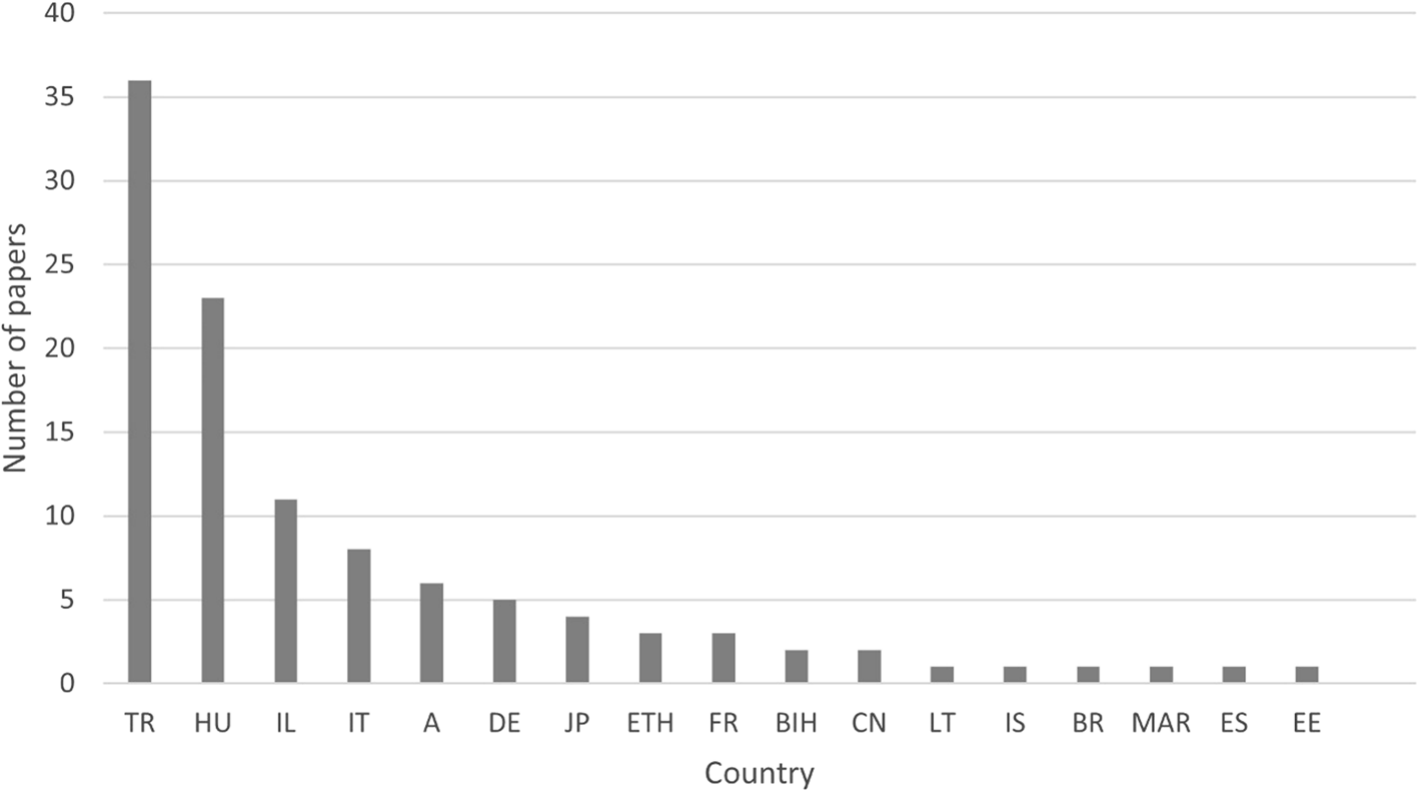
Total number of included papers by country. A: Austria; BIH: Bosnia; BR: Brazil; CN: China; DE: Germany; EE: Estonia; ES: Spain; ETH: Ethiopia; FR: France; HU: Hungary; IL: Israel; IS: Iceland; IT: Italy; JP: Japan; LT: Lithuania; MAR: Morocco; TR: Turkey

Most of the articles were written in Turkey (33.03%), followed by Hungary (21.1%), which was surprising considering the population size of the country (Fig. 2). A total of 10.09% of the articles originated in Israel.

**Fig. 3:**
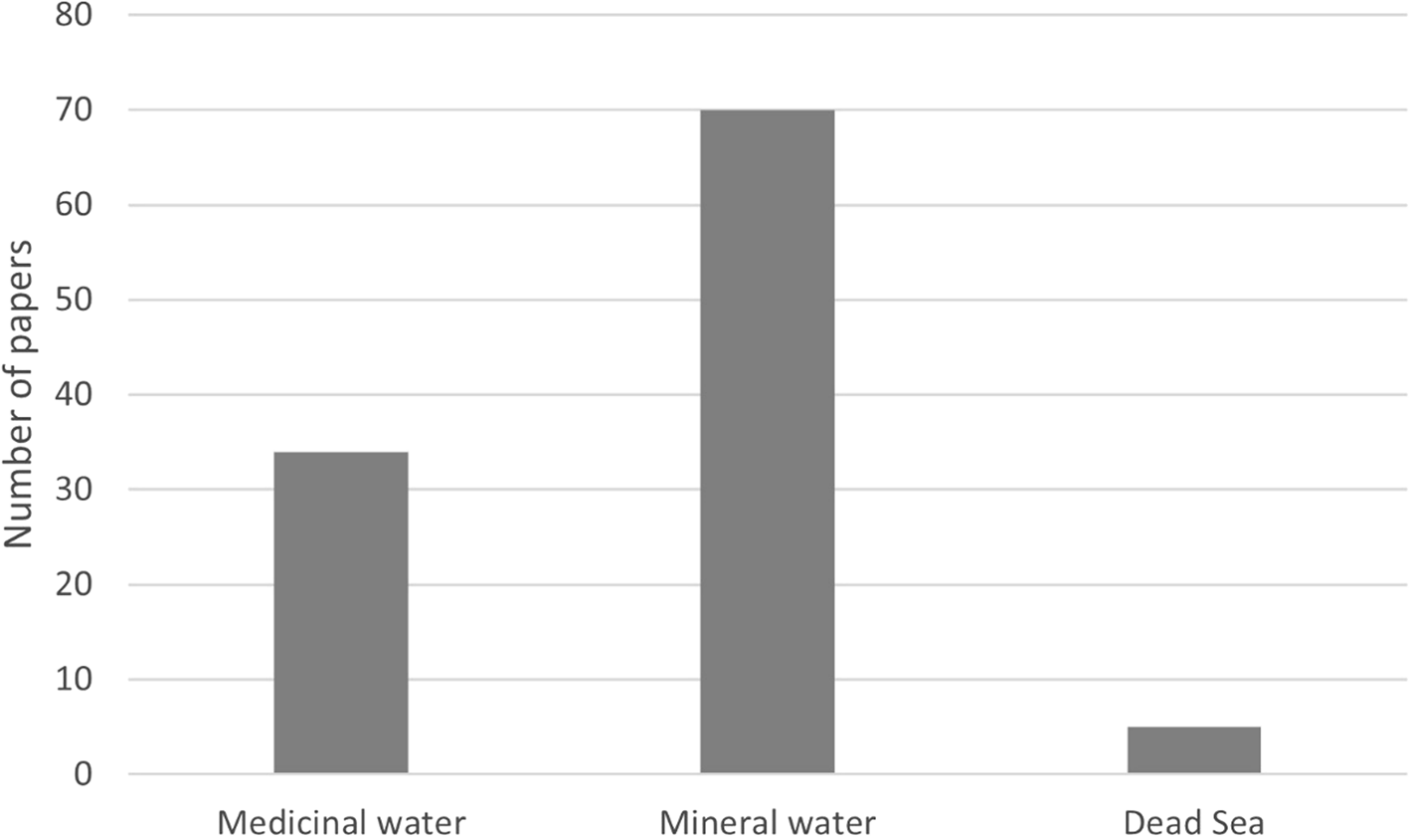
Distribution of papers by water type

Only 34 out of 109 articles (31.19%) examined the effects of scientifically proven natural medicinal water. There were 70 publications (64.22%) on natural mineral water and 5 (4.59%) on the Dead Sea (Fig. 3).

Among the included papers, Hungary published the most studies on the effects of natural medicinal waters, but the term ‘medicinal water’ also appears in German, Austrian, Italian, French, Chinese and Estonian papers.

**Fig. 4:**
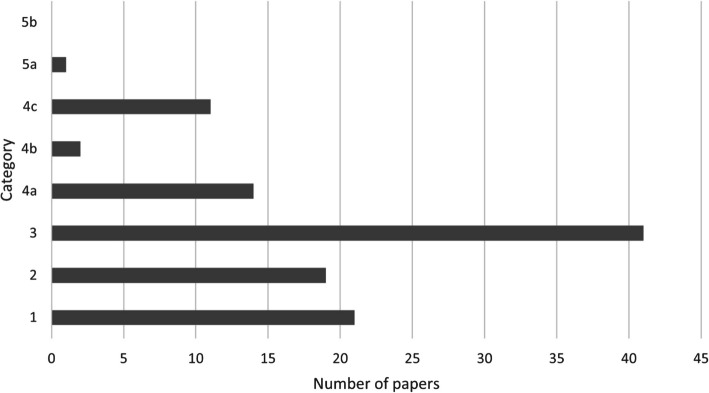
Total number of papers published in each category

Among the 109 publications, most belonged to category 3, accounting for 37.61% of the total. There were also several papers in categories 1 and 2. Thus, low-category publications constitute the largest group, with categories 1, 2, and 3 together accounting for 74.31% of the total. Among the higher categories (categories 4 and 5), category 4a had the highest number of articles, accounting for 12.84% of the total number of publications. No article was published in category 5b (Fig. 4). A chi-square goodness-of-fit test indicated that the distribution of publications across categories significantly deviated from a uniform distribution (*p* < 0.001), suggesting that certain categories, particularly category 3, contained a disproportionately high number of publications. To investigate the distribution of publications across different categories, we first assessed whether the data followed a normal distribution. However, the analysis revealed that the data significantly deviated from normality (*p* < 0.001, Kolmogorov–Smirnov test), with a skew towards lower-quality categories (1 and 2) and a concentration of publications in category 3. This indicated that the data was not normally distributed but rather followed a different pattern. Further analysis using the Poisson distribution model showed a good fit (*p* = 0.432), suggesting that the publication distribution can be described by a Poisson process, where the majority of publications fall within the middle categories (category 3), and both low (categories 1 and 2) and higher-category (categories 4 and 5) publications are relatively rare.

**Fig. 5:**
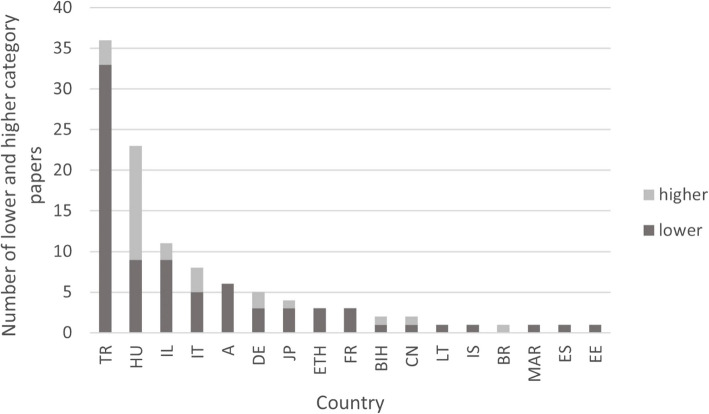
Proportion and number of lower (1, 2, 3) and higher (4, 5) category papers by country. A: Austria; BIH: Bosnia; BR: Brasil; CN: China; DE: Germany; EE: Estonia; ES: Spain; ETH: Ethiopia; FR: France; HU: Hungary; IL: Israel; IS: Iceland; IT: Italy; JP: Japan; LT: Lithuania; MAR: Morocco; TR: Turkey

Since most of the publications fell into category 3, the question arose as to which countries published them (Fig. 5). Turkey published the most articles in category 3, with more than half of the articles (23/41). Hungary followed by 6/41. Interestingly, Hungary published the most articles in higher categories (categories 4 and 5). To compare the distribution of high- and low-category publications across countries, we performed a Likelihood Ratio Test, which indicated a significant difference (*p* = 0.004). Post-hoc analysis of standardized residuals revealed significant differences for Turkey (z = -2.1) and Hungary (z = 3.3), suggesting that Turkish publications were in lower categories than expected, whereas Hungarian publications were in higher categories (Fig. 5). No other country exhibited such notable values.

**Fig. 6:**
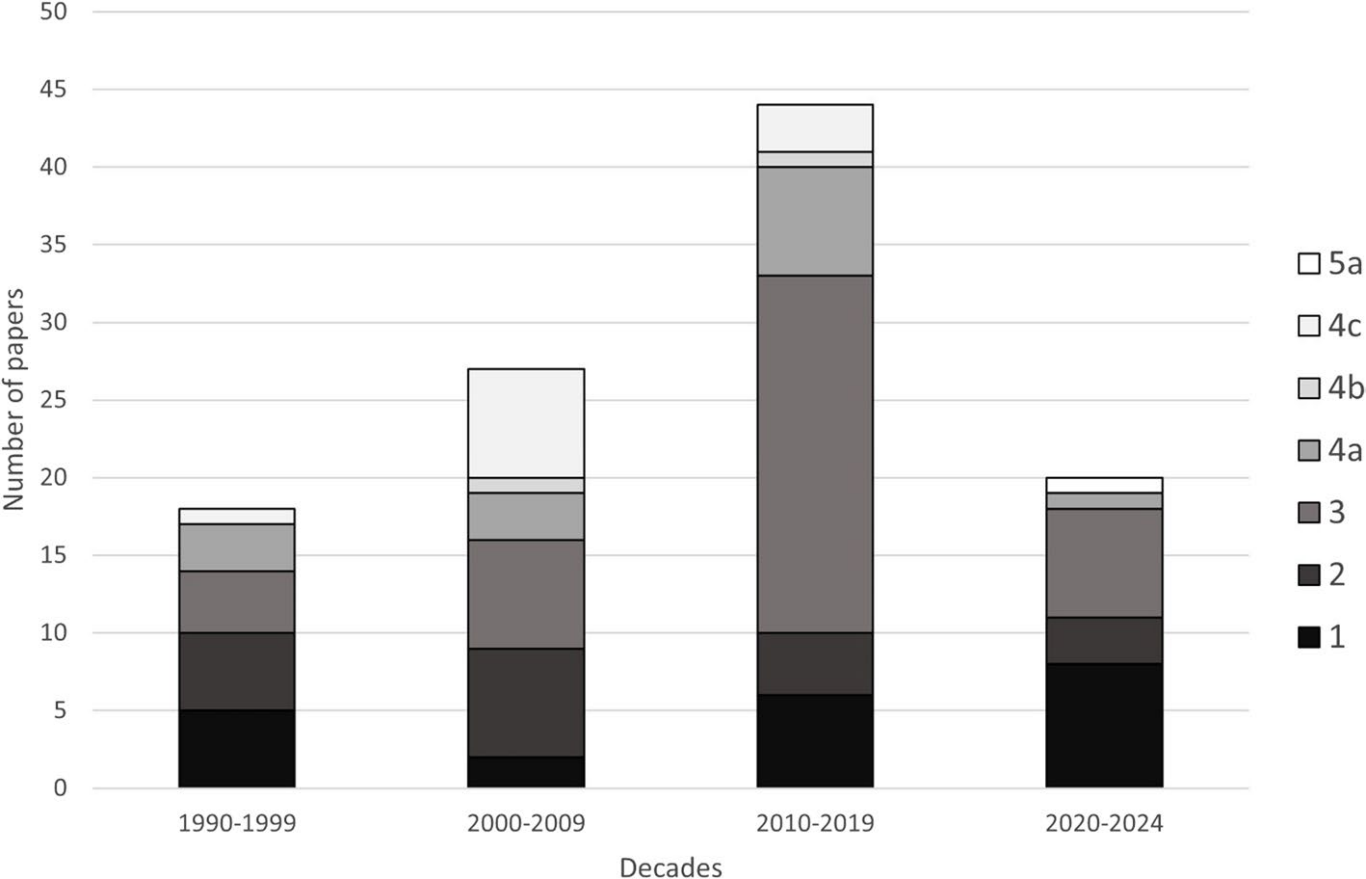
Proportion and number of all papers in all categories by decades

In Fig. 6 the data also revealed how the distribution of articles in each category changed from 1990 to the present. The last decade consisted of only five complete years (2020–2024); therefore, the results were analyzed with this consideration in mind. More publications have been produced in category 1 from 2010 to the present than in the two decades preceding it. In category 3 the number of publications increased considerably over the last full decade (2010–2019). Interestingly, category 4b, which used a single-blind setup, was not popular. Spearman’s correlation analysis revealed that, unexpectedly, the quality of publication categories has not shown a consistent improvement from 1990 to the present (*p* = 0.344; r = -0.091). Additionally, the Kruskal–Wallis test indicated that the median category ranking was highest in the second decade but lowest in the fourth decade (*p* = 0.018). This suggests that, on average, publications from the fourth decade fell into the lowest categories.

Logistic regression analysis assessing the distribution of publication categories by year revealed no significant increase in the proportion of articles classified as category 4 or higher over time (OR = 0.983; *p* = 0.476).

A detailed analysis of publication categories by year revealed no significant fit (*p* > 0.882) for any of the commonly used trend models, including Linear, Logarithmic, Inverse, Quadratic, Cubic, Compound, Power, S-Curve, Growth, Exponential, or Logistic. In other words, there has been no discernible trend in publication categories over the past 35 years.

A high percentage of publications (72.48%) did not use blinding. In 14.68% of the publications, the term “single-blind” was used incorrectly. In total, there were only two publications in which the trial was indeed single-blind. From 1990 to the present, only 12 out of 109 publications (11.01%) have attempted to use the double-blind setup (> 4c category), which is an important part of the ‘gold standard’ of EBM.

A total of 23.53% (8/34) of the medicinal water articles and only 5.71% (4/70) of the mineral water articles used the double-blind setup.

Randomization was used in 65.14% of the articles.

Researchers have tried to prepare control placebo water (category 4c) in 11 cases (without validation). To date, only one study has used validated placebo water (category 5a) [105].

The most popular journals seemed to be the International Journal of Biometeorology (with 17/109 publications), followed by Rheumatology International (10/109), Clinical Rheumatology (8/109), and Physikalische Medizin Rehabilitationsmedizin Kurortmedizin (9/109).

## Discussion

### Summary of evidence

To the best of the authors’ knowledge, this is the first scoping review summarizing the interventional study designs of clinical balneology trials on the effects of natural mineral/medicinal waters. The 109 studies identified in this scoping review revealed the number and types of interventional study designs and the number of open-label, single- and double-blind studies published between 1990 and February 2025. On the basis of the characteristics of the interventions, eight categories were created.

The results were not very favorable since most publications belonged to lower categories (categories 1, 2, and 3). It was expected that there would be more articles in the higher categories (categories 4 and 5) from 1990 to the present, as EBM is becoming more prevalent in science. However, this was not the case. There has been no discernible trend in publication categories over the past 35 years.

Most researchers did not even attempt the double-blind experimental design from 1990 until today. Not even those studies, where the color, scent, feel, pH, and buoyancy of the examined water were less significant. However, as stated earlier, the ideal setup would have been the single- or rather the double-blind setup because, in this case, the patients would not know whether they were in the experimental (medicinal/mineral water) or control (tap water) group. This raised an important question: is it certain that patients do not know which water treatment they were receiving? Experienced patients might have been able to distinguish between them. Therefore, the validation of placebo water is necessary [125].

Blinding was an interesting issue among the authors. From 1990 to the present, only 12 out of 109 publications (11.01%) used the double-blind design (categories 4c, 5a) [17, 21, 22, 49, 61, 62, 76, 77, 80, 105, 116, 123], and even fewer used the single-blind design (category 4b) (2/109) [92, 124]. A small number of balneology studies [48, 67, 101] misinterpreted the concept of single blindness. According to those studies, it did not matter who was blind in a single-blind study (e.g., ‘examining physicians were blinded’, ‘investigator was blinded’, ‘patients were instructed not to reveal their group assignment to the assessor’). Notably, in single-blind studies, the patient is blind [126]. When there is no blinding at all (categories 1, 2, 3, and 4a), the patient’s or investigator’s expectations (placebo effect) can affect the outcome to a nonnegligible extent, and false results can be obtained [11]. A very high percentage of publications (72.48%) completely lacked blinding. For example, in category 2, patients in the control group received only regular exercise as an intervention, and patients in the experimental group were treated with medicinal water. Clearly, blinding was not possible. However, in this case, the impact of the patients’ belief in medicinal water or regular exercise could not be eliminated. If this happens, the two placebo effects can be compared in the study. In a significant part of these open-label studies on natural medicinal/mineral waters, the authors claimed that they could not mask the trial due to the study design. Nevertheless, if two treatments under investigation cannot be made identical, more than one group can receive a placebo. This is the so-called double-dummy technique [127]. This technique could be used when placebo water, placebo medicine or injection is given. However, placebo exercise is difficult to perform.

It is also undeniable that creating the same conditions in a balneotherapy session for both the experimental and control groups is challenging. This situation was perhaps the most difficult in the case of the Dead Sea, where the therapeutic effects could not be examined under controlled conditions, especially if researchers set out to use at least a single-blind setup. (The problem could have been solved by using bathtubs.)

In the next part of the ‘gold standard’ of EBM, the presence of randomization will now be discussed. Randomization was not used in nearly 35% of the 109 publications.

As part of the ‘gold standard’ of EBM when a clinical trial is ‘controlled’, it is not necessarily sufficient in clinical balneology trials. This issue once again highlights the notion of blinding, since the type of treatment the control group receives is important. Among the 109 publications, only category 1 did not include a control group. This represented 19.27% of all studies.

Hungary is one of the leading countries worldwide that uses thermal water for medical purposes. As a small country, Hungary performs well internationally in the field of balneology, both in terms of the quantity and category of publications.

As this review focuses on the challenges of blinding in clinical balneological trials, it can now propose solutions to address them. Each natural medicinal water is a separate entity and a separate composition of active ingredients. For this reason, before comparing natural medicinal waters with existing, proven therapies for specific diseases, the authors deemed it necessary to decide whether these natural medicinal waters can stand on their own as medicine. This will require a validated placebo water trial as a first step to study if patients know what they are bathing in. Then a clinical trial with a validated placebo water control group is necessary as a second step (according to the ‘gold standard’ of clinical trials) to determine whether there is a significant therapeutic effect of the medicinal water compared with the placebo. Finally, as a third step, medicinal water can be compared with existing therapeutic options to test if it is better.

### Limitations

This scoping review has several limitations. The number of publications from the field of balneology was not as high as the authors expected. Unfortunately, many studies have investigated the effects of complex spa therapy, where the effects of medicinal water have been evaluated together with those of other spa treatments. In addition, several studies on artificially produced waters with healing effects, such as Dead Sea salt solutions, bath salts, Tibetan medicated bathing, Arjohuntleigh Parker Bath, thiosulphate bath, etc. also had to be omitted.

According to the results, only 31.19% of the publications examined scientifically proven natural medicinal waters. On the other hand, a high percentage of research (64.22%) has focused on natural mineral waters. While it is possible that these waters were certified medicinal waters in their countries, this was not mentioned in the papers.

The search strategy focused on balneotherapy and medicinal waters, so potentially relevant results that did not use these keywords were not captured.

Most of the articles were written in English, representing 88.07% of all the included articles. The databases used were unlikely to list all non-English-language publications. This is a potential source of error, as it is not known how likely non-English-language papers are to be included in the Medline, Embase and Cochrane databases.

Native English publications were missing from the included articles. One of the reasons for this might be that England does not have an abundance of medicinal water. With respect to the US, spas place more emphasis on exercise, reducing stress, lifting depression, and losing weight than on medical cures [128].

## Conclusions

This scoping review highlights significant methodological challenges in blinding within clinical balneological trials. The eight types of interventional study designs of the included articles provide a detailed picture. A key finding is that the majority of studies (74.31%) fall into lower-quality categories (1, 2, 3), where proper blinding and placebo control are absent, raising concerns about the validity of their conclusions. Despite the well-established standards of evidence-based medicine (EBM), only a small proportion of studies (11.01%) attempted to implement a double-blind design, and only one study used validated placebo water.

Geographical and temporal trends also indicate critical gaps in research quality. While Turkey has contributed the highest number of publications, these predominantly belong to lower categories, whereas Hungary has produced more high-category studies. Over time, there has been no clear improvement in study design, and in the most recent decade, publications have increasingly fallen into lower categories.

Given these findings, future balneological research should prioritize rigorous experimental designs, particularly by incorporating validated placebo water and double-blind methodologies. Without these improvements, the ability to draw reliable conclusions about the true efficacy of balneotherapy remains limited.

## Supplementary Information

Supplementary Material 1.

Supplementary Material 2.

## Data Availability

All data generated or analyzed during this study are included in this published article [and its additional files].

## Abbreviations

A: Austria
BIH: Bosnia
BR: Brazil
BT: Balneotherapy
CN: China
DE: Germany
EBM: Evidence-based medicine
EE: Estonia
ES: Spain
ETH: Ethiopia
FR: France
GE: Georgia
HU: Hungary
IL: Israel
IS: Iceland
IT: Italy
JP: Japan
LT: Lithuania
MAR: Morocco
TR: Turkey
